# Coevolution of longevity and female germline maintenance

**DOI:** 10.1098/rspb.2024.0532

**Published:** 2024-06-12

**Authors:** Julian Baur, Mareike Koppik, Uroš Savković, Mirko Đorđević, Biljana Stojkovic, David Berger

**Affiliations:** ^1^ Department of Ecology and Genetics, Uppsala University, Uppsala, Sweden; ^2^ Animal Ecology, Department of Zoology, Martin-Luther University Halle-Wittenberg, Halle (Saale), Germany; ^3^ Department of Evolutionary Biology, Institute for Biological Research “Siniša Stanković”, National Institute of the Republic of Serbia, University of Belgrade, Bulevar despota Stefana 142, Belgrade 11000, Serbia; ^4^ Institute of Zoology, Chair of Genetics and Evolution, Faculty of Biology, Studentski trg 16, 11000 Belgrade, Serbia

**Keywords:** life history, reproduction, parental care, mutation rate, offspring quality, germline

## Abstract

An often-overlooked aspect of life-history optimization is the allocation of resources to protect the germline and secure safe transmission of genetic information. While failure to do so renders significant fitness consequences in future generations, germline maintenance comes with substantial costs. Thus, germline allocation should trade off with other life-history decisions and be optimized in accordance with an organism’s reproductive schedule. Here, we tested this hypothesis by studying germline maintenance in lines of seed beetle, selected for early (E) or late (L) reproduction for 350 and 240 generations, respectively. Female animals provide maintenance and screening of male gametes in their reproductive tract and oocytes. Here, we reveal the ability of young and aged E- and L-females to provide this form of germline maintenance by mating them to males with ejaculates with artificially elevated levels of protein and DNA damage. We find that germline maintenance in E-females peaks at young age and then declines, while the opposite is true for L-females, in accordance with the age of reproduction in the respective regime. These findings identify the central role of allocation to secure germline integrity in life-history evolution and highlight how females can play a crucial role in mitigating the effects of male germline decisions on mutation rate and offspring quality.

## 1. Introduction

The interplay between growth, reproduction and survival is a central focus of life-history theory [1,2]. Trade-offs among these three fundamental aspects of an organism’s life play key roles in dictating the evolution of reproductive schedules, with cascading effects on organismal ageing and demography [3–5]. Indeed, some species invest heavily in early reproduction at the expense of longevity, while others prioritize growth and survival before reproducing, and these alternative strategies are expected outcomes of differences in mortality risk and age-related changes in the strength of natural selection [1,2,6].

Another, often less explicitly defined, dimension of life-history optimization involves the allocation of resources to protect the germline [7–9]. The integrity of the germline is essential for the transmission of genetic information to subsequent generations, and hence, the evolutionary consequences of germline maintenance strategies can be considerable both at the individual and population levels [10–12]. The costs of germline maintenance may be substantial [8], in part owing to the expensive repair machinery required to keep the rate of deleterious germline mutations at levels several magnitudes lower than the somatic mutation rate [13]. This sets the stage for trade-offs between the germline and other life-history traits that are governed by related processes, such as somatic maintenance and repair affecting longevity [14,15], or germline replication rate affecting sperm competition success [16–18]. Thus, costly maintenance of the germline may secure the genetic quality of future generations at the cost of reduced reproduction and/or survival of the parent [7–9].

Theories of ageing therefore do not only predict age-specific optimization of classic life-history traits but also the concerted evolution of processes aimed at protecting the germline. For example, the mutation accumulation theory of ageing [19] (hereafter ‘MA’) predicts a decline in germline maintenance past an organism’s reproductive peak owing to weakened selection and the accumulation of alleles with deleterious effects late in life. Indeed, ageing not only leads to somatic deterioration [20] but also negatively affects the maintenance of the germline [9], as shown, for example, by the fact that *de novo* germline mutation rates in primates seem to be foremost associated with advancing age [21–23]. Ageing can also be driven by antagonistic pleiotropy [24] (hereafter ‘AP’), whereby alleles with beneficial effects on germline maintenance at the peak of reproduction (e.g. via increased allocation of energy to germline repair) are favoured by selection despite potential deleterious side-effects on somatic maintenance [15], reproduction [14,16] and/or germline maintenance at other ages.

There is now growing evidence that germline decisions can be studied through a life-history lens. For instance, germline maintenance strategies can differ between environments that place different needs on maintenance versus reproduction [25–31] or between individuals that differ in their overall energy budgets and allocation decisions [14,16,18,32,33]. Interestingly, female animals possess the ability to provide maintenance not only of their own germline cells but also of male ejaculates and haploid male DNA, by synthesizing anti-oxidants in the reproductive tract to neutralize mutagenic reactive oxygen species, and by including mRNA and proteins in the oocyte to detect and repair DNA damage in the early zygote [34–36]. Additionally, females may also exert cryptic female choice of male sperm that compete for fertilization of eggs inside the female reproductive tract [37,38], which can serve as an additional barrier screening against male gametes of low genetic quality, even among sperm within an ejaculate from a single male [39,40]. This process bears similarity to apoptosis (controlled cell death) of damaged germline cells, which limits the passing on of deleterious mutations to offspring [36,41–44]. Here, we consider all these related processes (cryptic female choice, apoptosis, antioxidant defence and DNA repair) as alternative and non-mutually exclusive ways that females can exert germline maintenance in the broad sense. This female control over male gamete quality sets the stage for an intricate evolutionary dynamic, where care over transferred ejaculates and newly formed zygotes is the ultimate decision of the female, but the need for it might be dictated by the germline decisions of the male partner, or, as in human populations, his age [22,45,46].

**Figure 1.**
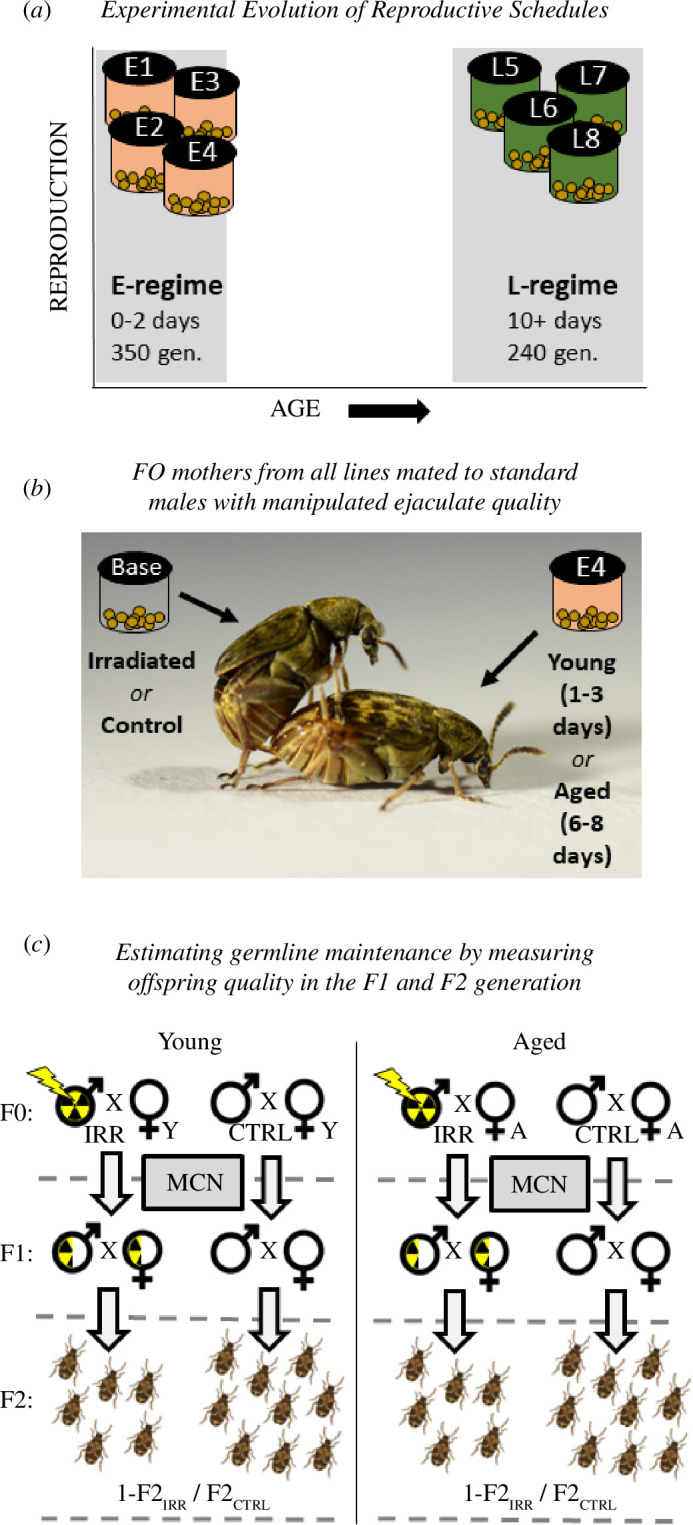
Experimental design. (*a*) The experiment included eight experimental evolution lines; four from the E-regime (early reproduction; salmon) and four from the L-regime (late reproduction; green). The E-regime was propagated by harvesting eggs laid after only 2 days of adult reproduction, whereas the L-regime was propagated by providing individuals that had aged 10 days with host seeds and harvesting the eggs. The E- and L-regimes had been kept for 350 and 240 generations, respectively, prior to the experiment. (*b*) Females from all lines, that were either young (1–3 days) or aged (6–8 days), were mated to standard males from the base population and allowed to lay eggs for 48 h. The males were either serving as controls or they had been irradiated to generate a damaged ejaculate. Offspring resulting from the matings to control males were counted to estimate the age-dependent reproductive schedules of the F0 females in the experiment. (*c*) F1 offspring were crossed within the line and treatment group (while avoiding inbreeding) using a middle-class neighbourhood (MCN) design to relax selection against induced (epi)mutations. Counts of emerging F2 adult offspring were used to assess age-specific female germline maintenance by comparing the proportional reduction in offspring quality in lineages deriving from either young and aged F0 females, mated with either irradiated or control males. Photo credit for panel (*b*): Mareike Koppik. Beetles in panel (*c*) were generated using BioRender.

Even though there now is a burgeoning understanding of the mechanisms that cause age-dependent variation in DNA repair and maintenance of the germline, little is known about the evolutionary potential of such mechanisms. In humans, for example, changes to environmental [47], pharmaceutical [48] and genetic [49] factors can increase longevity substantially within one or a few generations, and the peak of reproduction has in many populations changed drastically over the last century [50,51]. However, the long-term genetic consequences of lifespan extension depend on whether selection on reproductive schedules is coupled with concomitant changes in germline repair and maintenance, which remain unexplored. Here, we harnessed the power of long-term experimental evolution to investigate how the evolution of lifespan affects age-dependent germline maintenance in the seed beetle, *Acanthoscelides obtectus*. Using a set of lines that were established in 1989 and selected for early or late reproduction for more than 350 and 240 generations, respectively, we examined germline maintenance in young and aged females receiving male ejaculates with artificially inflated levels of free radicals and DNA and protein damage (figure 1). This allowed us to explore (i) whether female germline maintenance evolves in a concerted fashion with reproductive schedules and other life-history traits, and (ii) whether the AP [24] or the MA [19] theory of ageing best explains the evolutionary patterns observed.

## 2. Methods

### (a) Study species

The seed beetle, *A. obtectus* (Coleoptera; Chrysomelidae; Bruchidae) is a pest of stored legumes, foremost the common bean, *Phaseolus vulgaris* [52]. Females lay eggs either directly under or in close proximity to host seeds and the hatched larvae burrow into a seed where juvenile development takes place [53]. After pupation, adults emerge from the seed and typically become reproductively active within 24 h. Adult beetles are aphageous, meaning they do not need nutrition or water to be able to reproduce and finish their life cycle [52,54].

### (b) Experimental populations

The original base population, from which the experimental evolution lines were derived, was created by mass-mating wild-caught beetles from three locations in the area of Belgrade, Serbia, in 1983 [53]. Subsequently, the population was maintained on *P. vulgaris* in the laboratory for 3 years (27 generations) at a large population size (approx. 5000 individuals), until the experimental evolution lines were created in 1986. Experimental evolution was performed in dark incubators at 30°C. No food or water was offered to adult beetles.

During experimental evolution, one line was maintained under the same conditions as the base population (from here on referred to as the ‘base’ line); after 40 days of development, 400–500 adult beetles were chosen at random and provided with fresh host seeds and allowed to reproduce for the next generation. Each of the four replicate lines selected for early reproduction (E-lines) was created by taking 400 adults at random on the peak of adult emergence. To propagate the next generation, these adults were transferred to a new bottle containing host seeds ad libitum and were there allowed to reproduce and lay eggs for 48 h, after which all adults were removed. Another four replicate lines were selected for late reproduction (L-lines) by taking 100 newly emerged (0–24 h) adults every day for up to 10 days. Each of these subgroups was kept in a vial without seeds for 10 days. On the 10th day of each subgroup, all the surviving beetles of a given replicate line were added to a single bottle containing fresh host seeds to propagate the next generation (figure 1*a*).

The development time of E-lines has evolved to be 29 days, and the average virgin lifespan is around 13 days in aphagous conditions, while L-lines emerge after 32 days and virgin beetles live on average 26 days for females and 22 days for males [55]. The regimes have also diverged in other traits tied to rate-dependent life history, such as the day of first oviposition (E: 1 day after adult eclosion; L: 4 days after adult eclosion) [56], metabolic rate and body size [57]. Care was taken not to select directly on development time (by collecting adult beetles around the peak of emergence in each generation) or body size (by providing host seeds ad libitum. It can thus be expected that life-history evolution in the lines is a result of correlated responses to the applied selection on reproductive schedules. For more information on the maintenance of the experimental evolution lines, see [53,54].

### (c) Experimental design

The experiment was started in 2020, after 240 (L-lines) and 350 (E-lines) generations of experimental evolution. To estimate the evolution of age-dependent germline maintenance, we compared females of all lines mated to males when aged 1–3 days (young treatment) or 6–8 days (aged treatment) (figure 1*b*). Because we wanted to make inferences about evolved differences in biological ageing and correlated life history between the regimes, we compared E- and L-females at the same chronological age. We did not pick an older age than 8 days since E-females age rapidly, and we wanted to avoid any bias in the form of selective disappearance [58] of low-quality individuals in the E-lines. We note, however, that 6–8 days is not truly an old age for L-lines, but rather represents a relatively young reproductive age [53,55]. The comparison between E- and L-females of the same chronological age should thus be interpreted in relative terms.

All lines were reared in a staggered fashion by setting up several rearing containers of each line in the two generations prior to the F0 generation of the experiment so that the emergence of females would happen continuously during the experiment and females assigned to the two age treatments could be assayed simultaneously and mated to standard males from the same cohort (see the following text). We collected females on the day of their emergence, randomly assigned them to the young or aged treatment, and then isolated them in perforated 0.5 ml Eppendorf tubes and left them age. Females of both age treatments were then mated to young (1–2 days) virgin males from the base population in 60 mm petri dishes placed on heating plates kept at 30°C. Couples were observed to avoid multiple mating. Males were removed after mating behaviour had been observed, and females were allowed to lay eggs in a new 60 mm petri dish containing host seeds ad libitum for 48 h after the mating.

The base males were either untreated (controls) or had been exposed to a 10 Gy dose of γ-radiation, which causes substantial cellular damage to the male germline [43] and impairs male fertility in seed beetles [14,16,18]. The dose of 10 Gy was chosen based on our previous studies using similar experimental designs in the related seed beetle *Callosobruchus maculatus* [16,18,59], as well as another study on *A. obtectus* [60]. Hence, the female repair, maintenance and potential screening of damaged male gametes in each line could be assessed by comparing the offspring production in lineages stemming from females mated to control and irradiated males. γ-radiation produces free radicals with mutagenic properties and causes direct damage to DNA within sperm cells [43] and to proteins within the ejaculate [61]. Hence, our protocol captures a multitude of effects that occur naturally in male ejaculates [8,62], but that will have been greatly amplified by the irradiation treatment.

As our interest lies in capturing the effects of variation in female germline maintenance that transcend generations, we scored the fitness consequences of variation in female maintenance by counting the number of emerging F2 adults produced by all descending lineages. To achieve this, F1 males and females stemming from the same treatment group and line were paired (while avoiding sib-mating) and allowed to mate and produce F2 offspring in petri dishes with ad libitum host seeds (figure 1*c*). The emerging F2 adult offspring was counted to estimate each couple’s fecundity, which was used to estimate the germline maintenance of their F0 grandmothers (see §2d).

About half of the F0 couples produced no offspring (electronic supplementary material, table S1*a*). This was probably owing to two reasons. First, the mating latency of *A. obtectus* can be relatively long, and because the time between male irradiation and mating needed to be standardized (maximum of 30 min), many couples did not mate at this time. Second, even when mating was thought to have been observed, several couples did still not produce any offspring. Using a binomial model, we tested whether the incidence of zero fertility was related to any of the factors of interest in our experiment (i.e. irradiation treatment, selection regime and age treatment). However, despite the large sample size, no factor had a significant effect on infertility. Indeed, all effects including male irradiation treatment were very weak and non-significant (all *p* > 0.3), and the proportion of infertile couples was relatively equal across treatments (E: young ctrl = 0.43; young irr = 0.48; old ctrl = 0.59; old irr = 0.61. L: young ctrl = 0.50; young irr = 0.39; old ctrl = 0.48; old irr = 0.50). Hence, we carried out our downstream analyses based on the remaining data from the 830 F0 couples that produced offspring. Each line and age -class was represented by 10–20 females mated to control males, and 20–30 females mated to irradiated males. More couples were set up for the irradiated treatment group since the induced damage to male ejaculates was expected to generate more variance in offspring quality among lineages, requiring a larger sample size to result in the same precision of estimates as for the control lineages.

To reduce the effect of selection against epigenetic and DNA mutations induced by irradiation, we applied a middle-class neighbourhood (MCN) design [63] (figure 1*c*). The MCN design restricts each F0 couple to contribute the same number of F1 offspring for future study, irrespective of their overall productivity. We note, however, that F0 couples that produced less than four F1 offspring were not represented in the final data, and hence, selection on very strongly deleterious effects of irradiation could not be excluded by the MCN approach. For those F0 couples that did produce offspring, we aimed at propagating a single male and female from each couple, so that each F0 couple would replace itself. However, owing to variability in the timing of eclosion of F1 individuals, additional F0 couples fell out of the experiment as we did not want to age F1 beetles before setting them up in assays. These strict criteria resulted in 490 F1 couples being analysed for offspring production. Each line and age class was represented by approximately 11–27 and 5–15 F1 couples that originated from irradiated and control lineages, respectively (electronic supplementary material, table S1*b*).

The experiment was conducted in three temporal blocks, 3 days apart, corresponding to the three occasions on which we irradiated F0 males from the base population (electronic supplementary material, table S1*a*). In the F1 generation, when offspring productivity assays were set up, there was considerable overlap between these blocks owing to variable timing of egg laying and juvenile development time, so that combinations of F1 individuals stemming from all combinations of F0 dates were crossed. Hence, we did not include the effect of irradiation day in further analysis.

### (d) Statistical analysis

We analysed data using generalized linear mixed effect models implemented in the package MCMCglmm [64] for R [65]. Offspring counts were analysed assuming Poisson distributed errors. We used flat and uninformative priors for all models. We ran all models for 1.05 million iterations with an initial burn in of 50k iterations and a thinning factor of 500 to avoid autocorrelations, resulting in a posterior sample of 2000. P-values for all comparisons were calculated based on model posterior distributions, where the significance of main effects was calculated by comparing posteriors of marginal means of two given groups (e.g. overall difference in offspring production in irradiated and control lineages). We used ggplot2 for graphical illustration [66].

To provide an estimate of the extent of age-specific optimization of reproductive schedules at the current stage of experimental evolution, we first compared counts of offspring produced by young and aged F0 females of all E- and L-lines over the 48 h period, when mated to the standard base males belonging to the control (un-irradiated) treatment (*n* = 307). This model included female age treatment (young versus aged), evolution regime (E versus L), and their interaction, as fixed effects of interest. We also added the main effect of male age (1 or 2 days old) as a blocking factor. Replicate lines, crossed with age, were added as random effects (model summary in electronic supplementary material, table S2*a*).

To analyse differences in female germline maintenance, we compared the F2 offspring counts of control and irradiated lineages. This model included fully crossed fixed effects of evolution regime (E versus L), F0 female age treatment (young versus aged) and male radiation treatment (control versus irradiated). Note that, the three-way interaction between these effects tests our focal hypothesis that the E- and L-regimes have diverged in age-specific female germline maintenance. Replicate lines crossed with age treatment and radiation treatment were included as random effects (model summary in electronic supplementary material, table S2*b*). To provide an intuitive measure of germline maintenance for reporting effect sizes, we also calculated the relative reduction in fertility owing to unattended damage in the male ejaculate for each replicate line for young and aged females, according to Δζ_*i*_ = 1 − ζ_IRR, *i*_ / ζ_CTRL, *i*_, where ζ_IRR, *i*_ and ζ_CTRL, *i*_ are the numbers of F2 offspring produced by lineages deriving from F0 females of age treatment *i*, mated to irradiated and control males, respectively.

All data and code for analyses can be found here [67].

## 3. Results

### (a) Evolution of age-specific reproductive schedules

**Figure 2.**
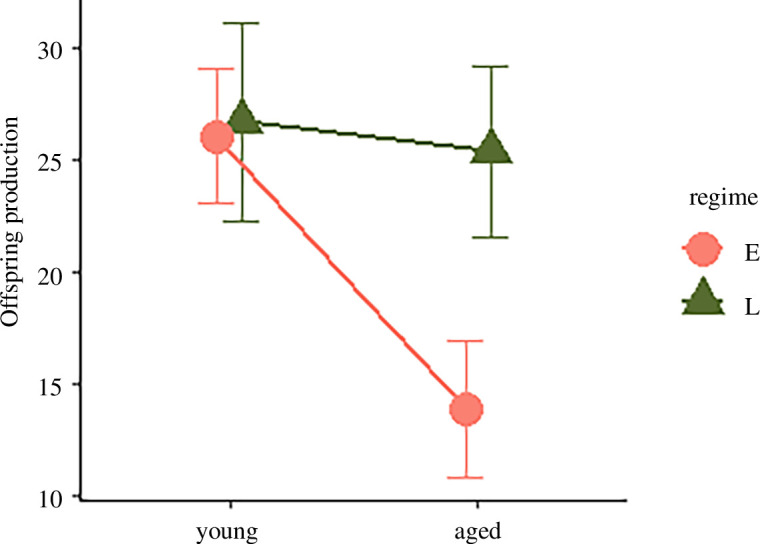
Evolution of female reproductive schedules. The number of F1 offspring produced by young and aged F0 females of the E- (salmon) and L- (green) regimes, during the 48 h of egg laying following mating to standard control males from the baseline. Shown are means and 95% confidence limits.

There was no difference in F1 offspring production between young E- and L-females mated to control males in the F0 generation (*p*_MCMC_ = 0.28). However, there were clear differences in ageing. As expected, aged females originating from the E-regime showed a sharp decline in offspring production (*p*_MCMC_ < 0.001), while no such effect was seen in the aged L-females. As follows, the interaction between regime and female age treatment was highly significant (*p*_MCMC_ < 0.001) and accorded with predictions (figure 2).

### (b) Coevolution of reproductive schedules and female germline maintenance

**Figure 3.**
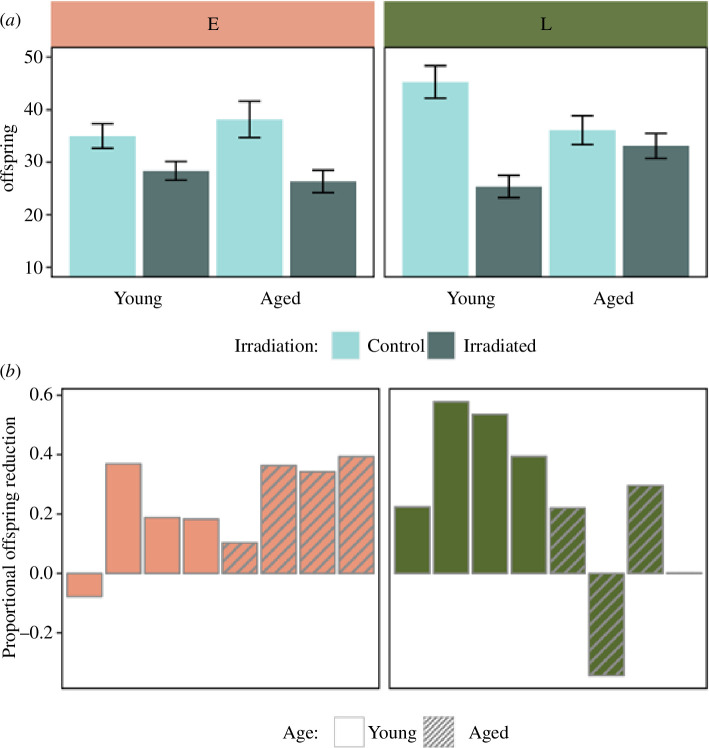
Evolution of age-dependent female germline maintenance. (*a*) F2 offspring production in lineages stemming from F0 grandmothers mated to either a control (light bars) or irradiated (dark bars) male. Grandmothers were either young (1–3 days) or aged (6–8 days) at mating and belonged to either the early reproducing (E: salmon, left panel) or late reproducing (L: green, right panel) evolution regime. Shown are means and standard errors. (*b*) Line-specific estimates of the relative reduction in F2 offspring production induced by the irradiation treatment in F0 males from the base population, calculated as Δζ*_i_* = 1 - ζ_IRR, *i*_ / ζ_CTRL, *i*_ , where ζ_IRR, *i*_ and ζ_CTRL, *i*_ are the number of F2 offspring produced by lineages deriving from F0 females of age *i*, mated to irradiated and control males, respectively. This estimate is thus inversely related to the efficiency of germline maintenance in F0 mothers. Plain and striped bars represent lineages originating from young and aged F0 grandmothers, respectively.

The irradiation treatment led to an overall decrease in F2 offspring numbers (effect of irradiation in L-regime: *p*_MCMC_ < 0.001; effect of irradiation in E-regime: *p*_MCMC_ = 0.002). Taken across both age treatments, there was no difference between evolution regimes in the effect of irradiation on offspring production (regime × irradiation interaction: *p*_MCMC_ = 0.74), providing no evidence for strong divergence in female germline maintenance overall. However, in terms of the age dependency, the two regimes seemed to behave very differently (regime × age × irradiation interaction; *p*_MCMC_ = 0.008; figure 3). This difference was owing to that the effect of irradiation was stronger in young, compared with aged, L-females (*p*_MCMC_ = 0.004), while the opposite pattern was observed for E-females (although the effect of age was not significant, *p*_MCMC_ = 0.35). These results confirm that female germline maintenance has evolved age-specific optimization.

## 4. Discussion

Here, we have shown that selection on reproductive schedules can lead to the evolution of age-specific female germline maintenance that mitigates deleterious effects caused by damaged male gametes. Such germline maintenance is a somewhat underestimated form of parental care with genetic consequences that can transcend generations. Our findings echo previous studies that have provided evidence that germline maintenance is costly and can trade off with life-history traits [14–16,32,68], emphasizing that germline maintenance should be considered a life-history trait in its own right [7,8,14,69–71].

### (a) Did germline maintenance evolve via mutation accumulation or antagonistic pleiotropy?

The observed evolution of ageing follows the general expectation that repair and maintenance in the female reproductive tract evolve to optimize function at peak reproduction. The mutation accumulation (MA) theory of ageing [19] predicts that E-lines should show efficient maintenance early in life, but a sharp decline in maintenance at old age, as old E-beetles have not been exposed to selection for over 350 generations. In L-lines, efficient maintenance of both young and aged females is expected, given that mature gametes have already formed at adult eclosion [60] and should need continuous maintenance. Indeed, the observed reproductive schedules of young and aged E- and L-females mated to control (unirradiated) males were qualitatively consistent with the MA hypothesis (figure 2). However, for female germline maintenance, E-females showed only a modest reduction with increasing age, and instead, it was young L-females that showed the lowest maintenance (figure 3). L- females have not been provided with egg-laying substrate at a young age for over 240 generations and egg-laying at this age thus represents a novel condition, which means that accumulation of mutations with conditional deleterious effects only at a young age cannot be excluded. However, while the dip in germline maintenance was larger in young (relative to aged) L-females compared to the dip in aged (relative to young) E-females, the E-regime had undergone more generations of (potential) accumulation of mutations with age-specific effects. Thus, the observed patterns of ageing of germline maintenance are not fully compatible with the MA hypothesis.

Another (not mutually exclusive) explanation for the evolved ageing trajectories is antagonistic pleiotropy (AP) [24]. Indeed, previous findings have shown that age-related changes in fertility and longevity in the E- and L-lines of *A. obtectus* seem to be a combined result of both MA and AP [72]. Pleiotropic effects of genes improving germline maintenance at a young age could lead to reproductive disadvantages at later ages [8,73] and/or reduced survival and longevity via trade-offs between somatic and germline DNA maintenance [8,15], and hence, would be disfavoured by selection in the L-regime. Interestingly, the pronounced dip in germline maintenance in young L-females is also mirrored by common observations of reduced fertility owing to reproduction too early in life across animal taxa [74]. Here, it is also worth underlining that the aged treatment in L-females actually represents a young reproductive age in this evolution regime, which likely corresponds to the onset of female reproductive investment during experimental evolution [53,54]. Hence, the E-females in the young treatment and L-females of the aged treatment should closely correspond to each other in terms of their biological age, and indeed, also show similar levels of reproductive output (figure 2) and germline maintenance (figure 3).

### (b) Male–female coevolution

The potential for females to care over male ejaculates already at the very onset of fertilization opens up interesting eco-evolutionary scenarios and the potential for sexual conflict over parental care, even in species traditionally assigned as having little to no care [18,68,75,76]. For example, in most species with internal fertilization, healthy females may efficiently compensate for poor ejaculates of older males, while the converse seems unlikely. This asymmetry should have implications for assortative mating by age. For example, the baseline germline mutation rate is several times greater in males compared with females in most animal taxa [71,77,78] and, at least in well-studied groups like primates, this bias increases with age [79,80]. Thus, mating decisions based on partner age are likely to have considerable consequences, and the form of maternal care studied here should be intertwined in those decisions.

Germline maintenance decisions can also depend on the overall condition of the individual and state-dependent allocation decisions between competing life-history demands [8,9,14,33], with implications for sexual selection theory and mate choice processes [16,18,81,82]. Viewing germline maintenance through a resource acquisition and allocation lens [83] suggests that optimal mate choice is contingent on not only choosers’ inferences of genetic quality in offspring based on the phenotype of their mating partner but also on how this phenotype influences changes to the genetic quality of its gametes. For example, in many polyandrous species, strong postcopulatory sexual selection (sperm competition) can favour compromised germline maintenance in males [16–18,69]. This suggests that males who are most successful in reproductive competition may sometimes pass on the greatest load of mutations to their offspring, questioning why females should prefer them in the first place [16,81,82]? Interestingly, however, optimality theory predicts that females may tailor care depending on the assessed state of male gametes [76], with the result that a male’s mutation rate is not independent of his female mate and her potential compensation for his short-comings, which can complicate interpretations of both age- and sex-biases in mutation rate [45].

Another way that females could influence the resulting quality of their offspring is via haploid selection through cryptic female choice of male sperm [40]. In this study, we defined female germline maintenance broadly, including both care of male gametes in the form of DNA repair and antioxidant defence, as well as screening against damaged sperm via cryptic female choice or damaged zygotes via apoptosis. Our study does not allow us to discriminate among these hypotheses, that are not mutually exclusive [42–44]. Yet, it is interesting to note that, while all these processes equate to germline maintenance and increased offspring quality from a female’s perspective, only female care improves male gamete quality, while female screening eliminates the male’s gametes. Therefore, the type of female maintenance may have different implications for male fitness, with the level of sperm competition within and between ejaculates of different males a predicted driver of these dynamics [84,85]. We do not know the extent of sperm competition in the E- and L-regimes, although it would seem likely that the number of different male mates per female might be higher in L-females owing to their prolonged lifespan. Nevertheless, we found no obvious differences between the evolution regimes in germline maintenance overall, or at the peak of early reproduction (young E-females versus aged L-females) (figure 3). Future research could investigate if females kept under high levels of sperm competition evolve increased cryptic female choice and if this is associated with correlated evolutionary responses in other aspects of germline maintenance.

In relation to male–female coevolution, we note that there is a possibility that our results could have been driven by an indirect effect of female reproductive ageing. If the F0 reference males in our experiment could assess the reproductive status of females, it is possible that males may have preferred females at their reproductive peak, and allocated resources in their ejaculates accordingly, resulting in higher offspring quality in young E- and aged L-females. However, we find this hypothesis unlikely owing to three reasons. First, in addition to requiring the ability of males to assess variation in female condition, there would also need to be sufficient fitness gains for this male allocation decision to evolve. We find this unlikely given the high-density scramble mating system under which the reference males have been evolving for at least 300 generations in the lab environment, and the relatively minor effects on female fertility caused by large variations in male ejaculate volume and quality across other species of seed beetle [86]. Second, the allocation decision would have needed to be very fast as most F0 males with offspring propagated to the F2 generation mated within less than 5 min after being introduced to the females, while processes that affect DNA repair and oxidant defence of male sperm typically occur over longer time frames [43], as does sperm maturation in the relative *C. maculatus* [16]. Third, this effect would also need to take effect in irradiated and not control lineages to generate the observed pattern (figure 3). Nevertheless, our current data do not allow us to completely rule out that this effect could have contributed to our results.

## 5. Conclusions

Can mechanisms that protect the germline evolve fast enough to keep up with rapid environmental and demographic change? This question is of importance for understanding long-term evolutionary responses that depend on both demographic parameters and the germline mutation rate. It is also a question tied to ongoing debates in human reproductive biology against the background of the increasing age at first reproduction in Western civilizations [87,88]. While assisted reproduction technologies may help to mitigate immediate age-related fertility declines associated with such changes, less attention is allotted to the evolutionary implications of ever-later reproduction in human populations. Our study on seed beetles provides compelling evidence that female care and/or screening of male gametes can evolve in response to selection on reproductive schedules. Germline maintenance peaked at the time of reproduction in E- and L-lines, and the observed evolution of ageing of the germline is compatible with some role for antagonistic pleiotropy in the underlying genes. This suggests that balancing selection on concerted life-history syndromes might be responsible for the maintenance of genetic variation in germline maintenance in the wild founding population, which likely contributed to the observed evolutionary response. Our study highlights the evolutionary importance of this often underappreciated type of maternal care, and we hope to stimulate further research aimed at elucidating the processes that govern the coevolution of germline maintenance, mate choice processes and other life-history traits.

## Data Availability

All data and code accompanying this study are uploaded to Dryad [67]. Supplementary material is available online [89].
